# Exploring diversification drivers in golden orbweavers

**DOI:** 10.1038/s41598-021-88555-3

**Published:** 2021-04-29

**Authors:** Eva Turk, Simona Kralj-Fišer, Matjaž Kuntner

**Affiliations:** 1 Evolutionary Zoology Laboratory, Institute of Biology, ZRC SAZU, Ljubljana, Slovenia; 2 Biotechnical Faculty, Department of Biology, University of Ljubljana, Ljubljana, Slovenia; 3 Evolutionary Zoology Laboratory, Department of Organisms and Ecosystems Research, National Institute of Biology, Ljubljana, Slovenia; 4 Department of Entomology, National Museum of Natural History, Smithsonian Institution, Washington, DC USA; 5 State Key Laboratory of Biocatalysis and Enzyme Engineering, Centre for Behavioural Ecology and Evolution, School of Life Sciences, Hubei University, Wuhan, Hubei China; 6 University of Ljubljana, Ljubljana, Slovenia

**Keywords:** Phylogenetics, Speciation

## Abstract

Heterogeneity in species diversity is driven by the dynamics of speciation and extinction, potentially influenced by organismal and environmental factors. Here, we explore macroevolutionary trends on a phylogeny of golden orbweavers (spider family Nephilidae). Our initial inference detects heterogeneity in speciation and extinction, with accelerated extinction rates in the extremely sexually size dimorphic *Nephila* and accelerated speciation in *Herennia*, a lineage defined by highly derived, arboricolous webs, and pronounced island endemism. We evaluate potential drivers of this heterogeneity that relate to organisms and their environment. Primarily, we test two continuous organismal factors for correlation with diversification in nephilids: phenotypic extremeness (female and male body length, and sexual size dimorphism as their ratio) and dispersal propensity (through range sizes as a proxy). We predict a bell-shaped relationship between factor values and speciation, with intermediate phenotypes exhibiting highest diversification rates. Analyses using SSE-class models fail to support our two predictions, suggesting that phenotypic extremeness and dispersal propensity cannot explain patterns of nephilid diversification. Furthermore, two environmental factors (tropical versus subtropical and island versus continental species distribution) indicate only marginal support for higher speciation in the tropics. Although our results may be affected by methodological limitations imposed by a relatively small phylogeny, it seems that the tested organismal and environmental factors play little to no role in nephilid diversification. In the phylogeny of golden orbweavers, the recent hypothesis of universal diversification dynamics may be the simplest explanation of macroevolutionary patterns.

## Introduction

Extant biological lineages exhibit a wide variation in species richness, ranging from hyper species rich lineages undergoing explosive radiation at one extreme to single representatives, sitting at the ends of long branches at the other. Heterogeneity in species richness is the product of the interplay between two fundamental macroevolutionary processes, speciation and extinction^1^. If speciation and cladogenesis are more frequent than extinction and lineage termination, the lineage diversifies and potentially radiates. If the opposite is true, the lineage eventually goes extinct^1^. While these mechanisms of diversity heterogeneity are understood, its drivers remain unclear.

Macroevolutionary literature generally assumes the differences in clade sizes result from accelerated or inhibited diversification (i.e. speciation minus extinction), which is in turn the consequence of a complex interplay between extrinsic factors (specific environmental conditions) on one hand, and organismal, intrinsic factors (e.g. states of particular traits) on the other^2–4^. Major tectonic events, emergence of new geographic barriers, changing patterns of wind and sea currents, shifts in local and global climatic conditions and the subsequent availability and variability of ecological niches are examples of closely intertwined extrinsic factors, influencing habitat-driven speciation and extinction^5–11^. In contrast, intrinsic factors are organismal traits (or their states) that correlate with, and presumably influence, taxonomic diversity. Body size has been repeatedly tested as a correlate of species richness due to ease of measurement and comparability of data among taxa. It has been found to correlate negatively with species richness in animals^12,13^, presumably because smaller animals have larger population sizes with access to more ecological niches^12^, lower energetic requirements^9,14^, shorter generation times^8^ and higher levels of mobility^8^.

Among animals, spiders provide an excellent platform for macroevolutionary studies, as they comprise a vast number of species with extremely diverse ecologies, life histories and evolutionary histories. Within spiders, golden orbweavers (Nephilidae) make an interesting subject for macroevolutionary analyses due to their well understood phylogeny and considerable age (estimated at 133 million years^15^), as well as their variation in phenotypes and species richness among the seven genera (1 sp. in *Indoetra* to 12 spp. in *Trichonephila*). Among their extreme phenotypes are extreme sexual size dimorphism (SSD), where females are up to 10 times larger than males, and web gigantism^15–18^. Here, we explore the macroevolutionary dynamics in nephilid spiders and search for evidence that would suggest diversification in this clade is driven by organismal and environmental factors. We infer the overall trends in speciation and extinction in the phylogeny and test several candidate factors, potentially responsible for the observed heterogeneity in diversity: geographical distribution (climate and landmass type), phenotypic extremeness and dispersal propensity.

**Figure 1:**
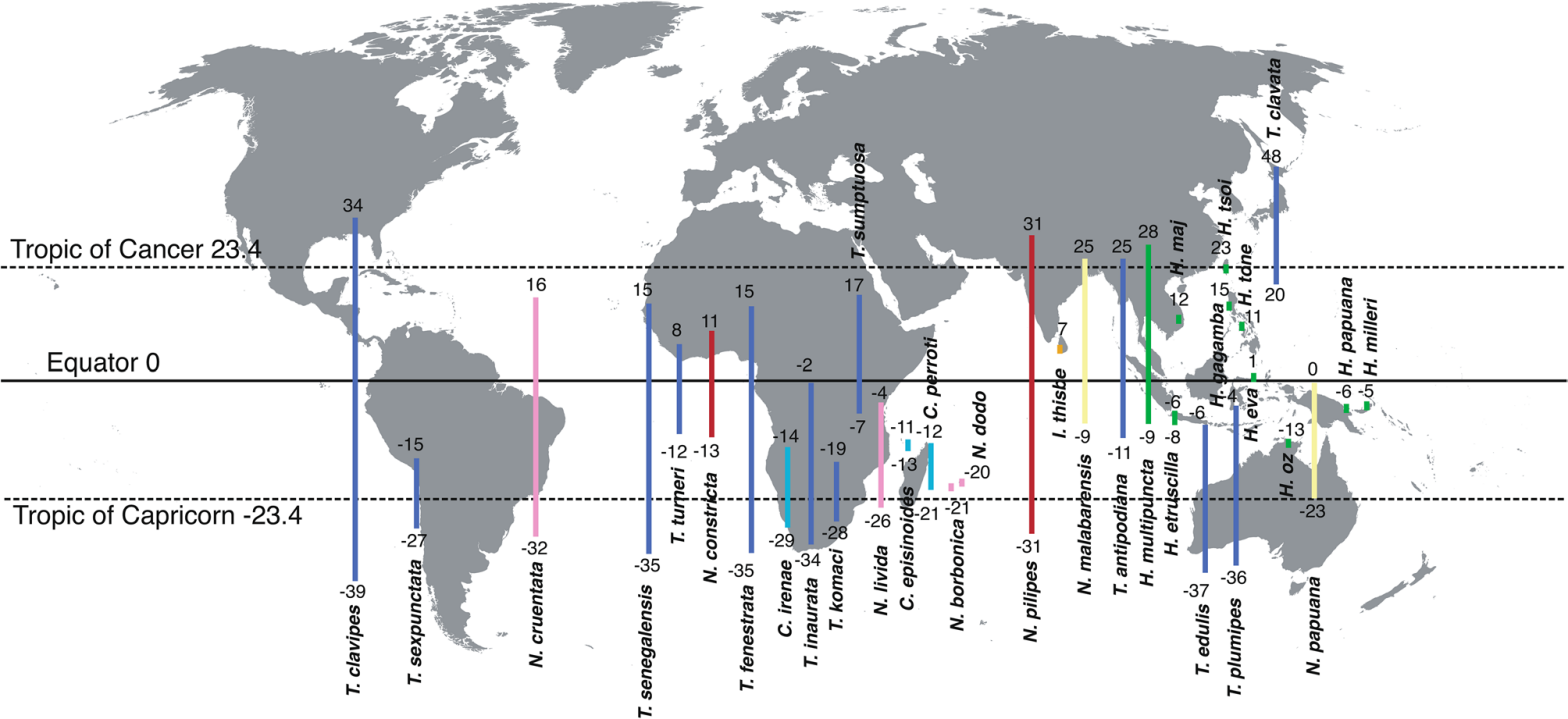
Approximate latitudinal ranges of 34 species of nephilid spiders included in the phylogeny. Latitudinal bars are colour-coded by genus: light blue – *Clitaetra*, dark blue—*Trichonephila*, red—*Nephila*, yellow—*Nephilengys*, orange—*Indoetra*, pink—*Nephilingis*, green—*Herennia*. Numbers denote latitudinal degrees of each species’ approximate range limits. The figure combines own distributional data with IUCN Red List data (https://www.iucnredlist.org/).

A latitudinal species richness gradient, with higher diversity in lower latitudes, is a well-known pattern found across animal taxa, including spiders^19,20^. This is also true for nephilids, where species are predominantly tropically distributed and seldom extend beyond the Tropics of Cancer and Capricorn (Fig. 1). We broadly test the latitudinal species richness pattern by evaluating the association between diversification and a binary factor of tropical versus subtropical distribution. We test island versus continental distribution as another binary environmental factor, under the premise that diversification is accelerated in clades with many cases of island endemism.

**Figure 2:**
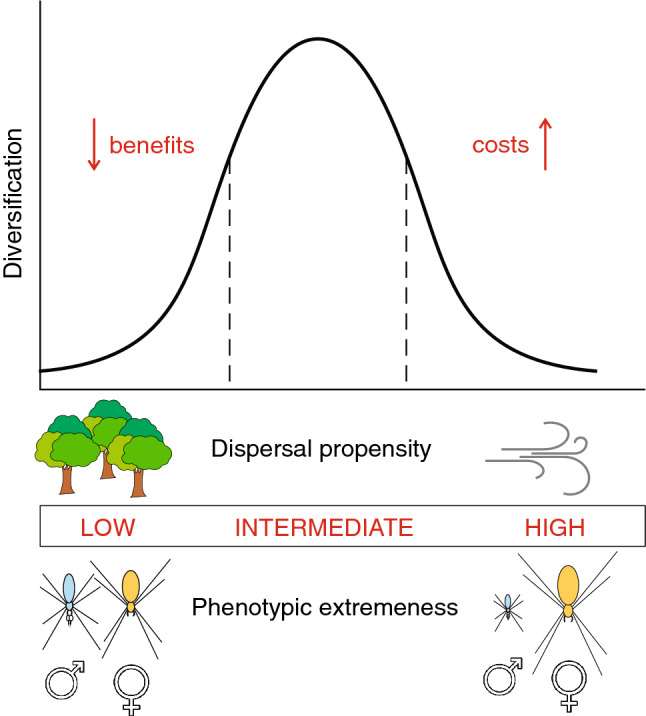
The hypothesised bell-shaped relationship between diversification and two continuous factors, dispersal propensity and body length sexual dimorphism. We test the prediction that intermediately expressed phenotypes will exhibit the highest rates of diversification.

For the two continuous traits, body length and dispersal propensity, we hypothesise a bell-shaped relationship between each trait and diversification—in other words, we expect to find the highest rates of diversification in genera with intermediate phenotypes and intermediate dispersal propensity (Fig. 2). For gigantism to evolve, it must provide certain fitness advantages, namely increased fecundity of large females^21^ and increased foraging success of large webs^22^. However, judging by the rarity of phenotypical extremeness and low species diversity of these lineages, extreme traits must also imply costs. Kuntner and Coddington^18^ suggest multiple possible disadvantages of giant female body size (increased predation risk, large nutrient demands), giant female web size (functionality constraints, larger numbers of kleptoparasites) and extreme SSD (genital mismatch, heterospecific mating, permanent sperm depletion). Traits that initially provide fitness benefits, but later in evolution lose their advantage and become costly (and potentially even cause lineage extinction) are termed ‘evolutionary dead end’ traits^23^, and extreme phenotypes in nephilids could be among them. We thus expect phenotypically extreme genera will exhibit lower rates of diversification, presumably due to elevated rates of extinction. On the other hand, genera with milder phenotypes do not enjoy the benefits of exaggerated phenotypes, like increased fecundity of giant females, which could likewise slow their diversification. Intermediately expressed phenotypes might thus prove to be the optimal state for maximising diversification potential.

Secondly, some nephilid genera, such as *Nephila* and *Trichonephila*, have wide distributions and disperse over large distances via ballooning, whereas others, such as *Nephilingis* and *Herennia*, exhibit low levels of dispersal propensity and maintain a narrow distribution^24,25^. Dispersal into a previously unoccupied area provides a spectrum of vacant ecological niches, a state previously shown to promote speciation in spiders^26^. Thus, inherent levels of dispersal propensity and behaviours related to dispersal might be influential intrinsic diversification factors, necessarily coupled with environmental conditions (i.e. extrinsic factors) allowing and limiting ballooning, such as changes in global wind patterns and emergence of geographical barriers restricting air currents. According to the intermediate dispersal model^27–29^, poor dispersers maintain a narrow distribution and only occupy new space (and consequently speciate) rarely. At the other extreme, excellent dispersers occupy large ranges, but also successfully maintain gene flow across them, inhibiting population fragmentation and, with it, speciation. Highest levels of diversification should thus be found in intermediate dispersers (Fig. 2) that are able to overcome dispersal barriers occasionally, but not often enough to maintain gene flow with neighbouring populations.

Additionally, we explore whether web type, another manifestation of extreme phenotypes in nephilids and an example of their extended phenotype, influences diversification. Types of webs differ between nephilid genera, but not within them. If web type has an effect on the rates of speciation and extinction, the underlying reasons may be related to differences in captured prey type, prey quantity and protection from predators each web type provides. The methods, results and discussion relating to this analysis are available in Supplementary Information online.

## Results

### Accumulation of lineages through time

**Figure 3:**
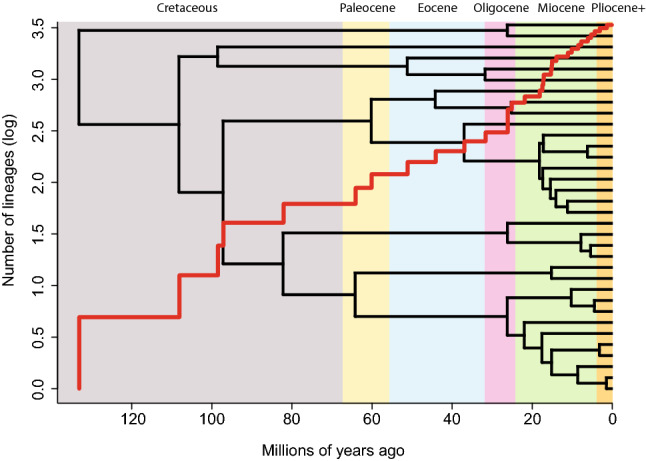
A lineage-through-time plot, showing species number accumulation (in red) on a logarithmic scale over the nephilid phylogenetic topology.

The semi-log lineage-through-time plot on a nephilid phylogeny shows a relatively linear overall trend, without an obvious upturn towards the present (Fig. 3). The gamma statistic score (γ = 0.908, p = 0.36) and MCCR test (p = 0.26) do not imply an early burst in diversification, followed by its decrease. Instead, the accumulation of new lineages is denser towards the present (Fig. 3).

### Macroevolutionary rate inference

**Figure 4:**
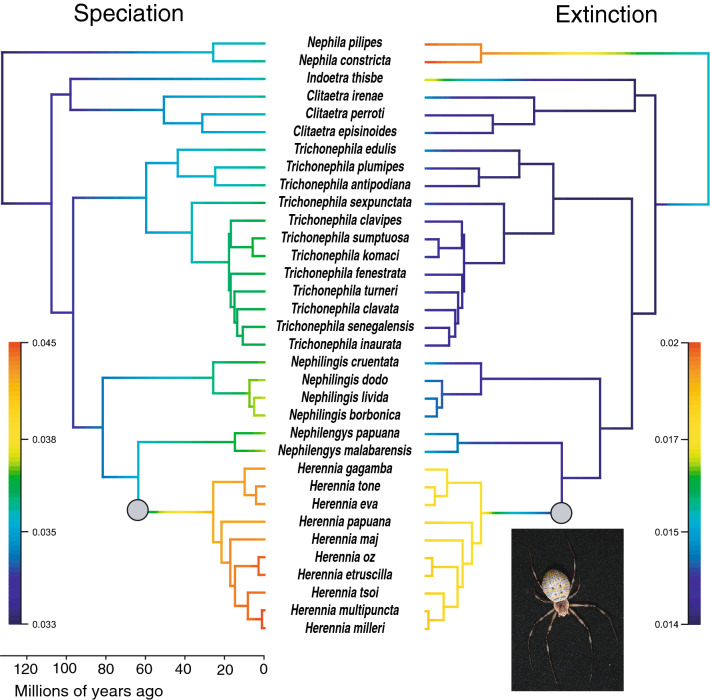
Macroevolutionary rate inference using two methods. BAMM phylorate plots show speciation and extinction rate dynamics in the nephilid phylogeny. Colours denote relative intensity of speciation and extinction along branches. Grey dots signify the location of a shift in diversification rate, inferred by MEDUSA. It detected increased diversification in the genus *Herennia* (visually presented by *H. eva*; own photograph) relative to the rest of the phylogeny.

BAMM analysis detects no distinct shifts in nephilid speciation or extinction rate dynamics. Similarly, the rate-through-time plots show no cumulative trend in either process across the clade’s evolutionary history (see Supplementary Figs. S1 and S2 online). However, the mean phylorate plots for both rates do display differences in rate dynamics across the phylogeny (Fig. 4). The highest rate of speciation is recovered in *Herennia*, but in other parts of the phylogeny, speciation rate is relatively uniform, showing an overall increase from the root to the present. On the other hand, a strongly accelerated extinction rate is recovered in the species poor genus *Nephila*, and to a lesser extent in *Indoetra* and *Herennia* (Fig. 4). The remaining genera exhibit much lower, uniform extinction rates. RevBayes corroborates heterogeneity in macroevolutionary rates across nephilids, with an inferred increase in diversification in *Herennia* and *Trichonephila* (see Supplementary Fig. S3 online). Lastly, MEDUSA indicates one shift in diversification rate, placed at the base of *Herennia*, at approximately 65 Ma (Fig. 4). In this clade, it detects significantly greater diversification (r = 0.08) relative to the background rate (r = 0.02).

### Correlating candidate factors to macroevolutionary rates

We used SSE-class models to test whether nephilid diversification correlates with the states of selected organismal and environmental factors. For both selected binary environmental factors, continental vs. island distribution and tropical vs. subtropical distribution, there is no significant difference between full models and those with constrained speciation, extinction and transition rates from one state to the other (Table 1). This result implies no difference in the three macroevolutionary processes between species from different environmental conditions. The only marginally significant result (p = 0.06) suggests a difference in speciation rate between tropically and subtropically distributed lineages, with higher rates in the tropics. Similarly, there were no improvements in model fit compared to basic BiSSE (null model) when rates were allowed to change at 26 million years ago (mya) for island versus continental (p = 0.79) and at 45 mya for tropical versus subtropical distribution (p = 0.88). When each rate was individually allowed to change at the time switch point, there were likewise no significant improvements of model fit (Table 1).

**Table 1:** ANOVA comparisons of SSE-class models.

BiSSE						
	Continental vs island distribution			Tropical vs subtropical distribution		
Model	lnLik	AIC	Pr( >|Chi|)	lnLik	AIC	Pr( >|Chi|)
Full model	− 166.70	345.40		− 163.85	339.70	
λ_1_ = λ_0_	− 166.95	343.90	0.48	− 165.59	341.17	0.06*
μ_1_ = μ_0_	− 166.72	343.44	0.84	− 164.33	338.67	0.33
q_10_ = q_01_	− 167.58	345.17	0.18	− 164.09	338.18	0.49

Time-dependent BiSSE						
	Continental vs island distribution			Tropical vs subtropical distribution		
Model	lnLik	AIC	Pr( >|Chi|)	lnLik	AIC	Pr( >|Chi|)
Full model (no constraints)	− 165.12	354.24		− 165.49	354.99	
Free λ	− 165.28	346.56	0.99	− 165.95	347.90	0.92
Free μ	− 165.66	347.33	0.90	− 166.08	348.16	0.88
Free q	− 166.08	348.16	0.75	− 166.16	348.31	0.86
Null model (all constrained)	− 166.70	345.40	0.79	− 166.70	345.40	0.88

QuaSSE						
	Female body length			Male body length		
Model	lnLik	AIC	Pr( >|Chi|)	lnLik	AIC	Pr( >|Chi|)
Constant	− 272.19	550.37		− 189.55	385.11	
Linear	− 272.18	552.35	0.88	− 188.89	385.78	0.24
Sigmoidal	− 272.18	556.35	1.00	− 188.91	389.82	0.73
Hump	− 271.98	555.97	0.94	− 188.89	389.78	0.72
Drift.linear	− 272.08	554.17	0.90	− 188.88	387.77	0.51
Drift.sigmoidal	− 272.14	558.28	1.00	− 188.87	391.73	0.85
Drift.hump	− 270.86	555.71	0.62	− 188.84	391.68	0.84

	Body length SSD			Dispersal propensity (AOO)		
Model	lnLik	AIC	Pr( >|Chi|)	lnLik	AIC	Pr( >|Chi|)
Constant	− 206.62	419.24		− 244.07	494.13	
Linear	− 206.41	420.81	0.51	− 244.05	496.10	0.86
Sigmoidal	− 206.19	424.39	0.84	− 243.07	498.15	0.58
Hump	− 205.21	422.41	0.42	− 243.13	498.26	0.60
Drift.linear	− 206.33	422.65	0.75	− 243.73	497.47	0.72
Drift.sigmoidal	− 205.98	425.97	0.87	− 242.73	499.47	0.62
Drift.hump	− 205.00	423.99	0.52	− 242.83	499.66	0.65

BiSSE tests for an effect of two binary traits on speciation (λ), extinction (μ) and state transition (q), with the time-dependant variant additionally allowing for rate change at a specified point in time. Each model is tested against the full model. QuaSSE fits the relationship between speciation and four quantitative traits to four alternative shapes. The last three models additionally allow for drift (directional trend). The asterisk signifies a marginally significant p value (0.05 < p < 0.1).

Lastly, we tested which function, if any, describes the relationship between diversification (with extinction held constant) and continuous traits of female body length, male body length and body length SSD as examples of extreme phenotypes, and dispersal propensity. None of the models are even marginally statistically supported for any trait (Table 1), suggesting the evolution of the selected continuous traits is unrelated to diversification in nephilids.

## Discussion

Nephilid evolutionary history has been a subject of much scientific inquiry, particularly due to the striking variety of phenotypes and species richness, relative to the family’s size. Ours is the first examination of the dynamics of two main macroevolutionary processes, speciation and extinction, on a robust nephilid phylogeny. Using a suite of different approaches to inferring diversification dynamics, we test potential organismal and environmental drivers of the rates’ heterogeneity and find no clear support for the hypothesized influence of extreme phenotypes and dispersal propensity on diversification of golden orb weavers.

### Accumulation of lineages through time

Summarizing the phylogeny, the semi-log LTT plot shows a roughly linear trend, with only a hinted upturn in the last 30 million years, but with an accelerated accumulation of new lineages towards the present (Fig. 3). A linear trend is in accordance with a pure-birth model of evolution (further supported by the gamma and MCCR test statistics), suggesting negligible extinction throughout nephilid evolutionary history. An upturn in the LTT plot, on the other hand, could be interpreted as the ‘pull of the present’—an apparent increase in diversification towards the present, that is in fact the consequence of a non-zero extinction rate. Because younger clades have not yet had time to go extinct, this could inflate recent diversification rates^30–32^. If so, the pattern detected here could indicate that our studied lineage may have recently broken its diversification stasis, entering a phase with livelier speciation and extinction. The presence or absence of an upturn in the plot towards the present is ambiguous, however, and the lack of a clear pattern might be a consequence of the small absolute number of taxa in the phylogeny.

### Macroevolutionary rate inference

In BAMM rate-through-time plots, the very slight increase in speciation and a completely flat trend in extinction are a surprising result, further corroborating the pure-birth model of evolution. The failure of BAMM to recover any rate shifts is also unexpected, although Kodandaramaiah and Murali^33^ show that it underestimates rate shift numbers in small phylogenies, such as this one, and recommend a careful interpretation of a zero rate shift result in such cases. Interestingly, despite the lack of rate shifts, BAMM recovers large differences in rates along different branches (Fig. 4). *Herennia* is the only clade that stands out by speciation rates, and MEDUSA even recovers a rate shift at its base. This may be related to the high occurrence of island endemism in the genus, its persistence within a single biogeographic region, and thus speciation by vicariance^34,35^.

On the other hand, BAMM infers the largest extinction rates in the genus *Nephila*, potentially pointing towards the ‘dead-end’ nature of *Nephila*’s life history^18^. Moreover, both *Nephila* and *Indoetra*—a monotypic genus also exhibiting heightened levels of extinction, are genera with only one or two extant species, sitting at the tips of very long branches. One can speculate these branches may have contained other species in the past that went extinct during the course of their long evolutionary histories—be it due to extreme phenotypes or other reasons. Accelerated extinction is also recovered in *Herennia*—a relatively young clade, where extinction cannot be attributed to the same causes. Here, we hypothesise it relates to the species’ limited ranges, often confined to single islands, and consequently smaller population sizes, making them more susceptible to extinction.

Branch-specific diversification analysis with RevBayes corroborates livelier diversification in *Herennia*, but also identifies *Trichonephila* as a rapidly diversifying clade. BAMM, on the other hand, recovers no exceptional rates for either speciation or extinction in *Trichonephila*, despite the clade’s comparatively large extant diversity. This discrepancy in the results between the two closely related methods may be a manifestation of the differences in rate calculation, referred to in the Methods, and implies the importance of method selection in such analyses.

### Phenotype extremeness and dispersal propensity do not affect macroevolutionary rates

Neither of the two binary traits tested with BiSSE and its time-dependent variant reveal a strong correlation with diversification, although tropical versus subtropical distribution does show a marginally significant relationship with speciation. This result is not surprising, as the tropics do in fact host a much larger taxonomic diversity of nephilids than the subtropics (Fig. 1). On the other hand, a lack of correlation between speciation and island distribution is unexpected, as some genera, especially *Herennia* and *Nephilingis*, contain island endemic species. This is closely related to the idea of dispersal propensity as a major intrinsic factor of diversification, in that these species are such poor dispersers they do not maintain gene flow even with adjacent islands and should exhibit speciation by vicariance.

Our main prediction was that phenotype extremeness and dispersal propensity are correlated with diversification through a bell-shaped function, suggesting intermediate phenotypes and intermediate dispersal propensity promote diversification the most. Interestingly, none of the tested types of relationship between speciation rates and quantitative trait values received statistical support for any of the four tested factors, dispersal propensity, female and male body length, and their quotient, body length SSD. They could thus either be in a type of relationship not tested by QuaSSE, or, more likely, be independent of each other. The results also imply that speciation is not linked to any direct correlates of the tested factors. Despite these results, we see heterogeneity in speciation rates recovered by BAMM. The tested traits seem not to be the drivers behind these differences, but other factors may exist that play this role.

### Alternative explanation: universal diversification law?

A recent paper by Diaz et al.^36^ argues that too much emphasis has been placed on identifying biotic and abiotic factors as drivers of macroevolutionary dynamics. Instead, they suggest a biological generality, that the youngest clades diversify (i.e. speciate *and* go extinct) the fastest and the oldest clades the slowest, regardless of the organism, its biogeography or ecology. The authors speculate the reason for this ‘universal diversification law’ is the concentration of speciation and extinction events in specific points in time, with long intervals of macroevolutionary stasis in between^36^. Whether these concentrations happen due to regular fluctuations in global climate, complex ecosystem dynamics or some other factor remains unanswered.

While the concentrations of speciation and extinction events, proposed by Diaz et al.^36^, might not be as obvious at the scale of our small, comparatively young phylogeny, it is interesting to note that lively branch splitting occurs in the last 20 million years (i.e. the Neogene) in all nephilid genera except *Clitaetra* and *Indoetra*. Before that, branch splitting is sparse and evenly distributed in time, resembling a period of diversification stasis. In contrast to this pattern of relatively recent lively cladogenetic events, it seems that earlier nephilid evolutionary history had more shifts among biogeographic realms. Namely, Turk et al.^34^ reconstructed much more frequent migration across biogeographic regions in the Paleogene and Cretaceous compared with the Neogene, when each genus diversified almost exclusively within a single biogeographic region. This is especially notable in a subclade of *Trichonephila*, which colonized the Afrotropics in the beginning of the Neogene and continued rapid diversification predominantly within Africa. In this case, empty ecological niches could have acted as a major extrinsic driver of adaptive radiation, although this remains to be tested.

### Limitations in methodology

Heterogeneity in diversification across space and time has long been a subject of interest in evolutionary biology, but research has long proven difficult due to poorly reconstructed phylogenies lacking time calibration, scarce fossil records and a lack of specialized statistical tools. Advancements in molecular phylogenetics enabled the production of robust phylogenies and with them the development of superior macroevolutionary rate estimation methods (reviewed in e.g.^32,37,38^). However, there is an extensive ongoing debate in macroevolutionary literature on how and when macroevolutionary rates should be estimated (e.g.^39^). This is especially true for extinction rate estimation in clades with no fossil records and with non-uniform diversification rates, like ours (see “Methods”). The field is far from reaching a consensus, with some work disputing the validity of rate estimation altogether^40^.

It is important to acknowledge a crucial limiting factor in our analyses, the relatively small size of the phylogeny. Several studies have shown empirically that the number of terminals in a lineage can profoundly influence the performance of an array of macroevolutionary analyses, where small phylogenies show limits in statistical power and accuracy^33,41,42^. Some taxonomic groups, like nephilids studied here, have relatively low species numbers, but their macroevolutionary rates nonetheless remain an interesting object for research. One might speculate that the lack of support for our main hypotheses is at least in part due to the small absolute number of taxa, making the relationship with diversification difficult to detect. We hope to see advancements in this field in the future, providing adequate macroevolutionary methodology for smaller taxonomic units.

## Conclusion

While there might be biotic and abiotic factors that further stimulate or inhibit diversification, species emergence, persistence and demise are complex processes, requiring the right conditions at the right time to unfold. A universal pattern of diversification might indeed explain why we fail to find correlation between any of the tested traits and diversification—it is simply not as dependent on organismal and environmental traits as predicted. Considering the same patterns of diversification are found across the tree of life, results of studies like ours might be best explained in the simplest way possible.

## Methods

All diversification analyses were performed in *R* v.3.5.0^43^, on a time-calibrated, ultrametric phylogeny after Turk et al.^34^ (see Supplementary Data online). This phylogeny includes 34 out of 40 currently described nephilid species, meaning 85% taxon sampling.

### Accumulation of lineages through time

Using the R package *phytools* v.0.7-70^44^, we produced a lineage-through-time (LTT) plot to visualise species accumulation through time, calculated the gamma (γ) statistic and ran the Monte Carlo constant rates test (MCCR) (both after^45^). Both statistics test whether observed diversification in a phylogeny deviates from diversification rates, expected in a pure-birth model, with the MCCR test additionally accounting for incomplete taxon sampling. A significant value of both statistics is generally interpreted as recent deceleration in diversification rate, implying an early burst in diversification^46^. Although a popular method of detecting potential early bursts, it has been criticized for being overly sensitive to recent changes in diversification rates compared to those in the early history of the tree, regardless of the tree’s size and completeness of sampling^46^.

### Macroevolutionary rate inference

We inferred overall macroevolutionary dynamics in the phylogeny with BAMM v.2.5.0 (Bayesian Analysis of Macroevolutionary Mixtures)^47^. It uses reversible jump Markov Chain Monte Carlo to survey and assess a large number of potential diversification models for a given phylogenetic tree, each with a unique configuration and intensity of macroevolutionary processes^47^. The main aim of BAMM is to identify points of rapid change in rate intensity, known as rate shifts. An important benefit of this method is that it does not assume the rate of the processes to be constant through time, but to behave dynamically^47^.

We used BAMM to infer rates of speciation and extinction. Speciation (λ) and extinction (μ) priors were set using the ‘setBAMMpriors’ function in the R package *BAMMtools* v.2.1.7^48^. Due to the relatively small phylogeny, the expected number of rate shifts was set to 1. Additionally, incomplete taxon sampling was accounted for in the control file by specifying which genera lack complete species representation and to what extent. BAMM was set to run for 10 million generations on four MCMC chains, sampled every 1000 generations. We guaranteed chain convergence by confirming that effective sample size (ESS) values were > 200. Results were analysed using *BAMMtools* and visualised in the form of rate-through-time plots and mean phylorate plots, which display varying rates by colour-coding branch segments.

Despite its popularity, BAMM has seen criticism regarding its reliability (^49^, but see^50^). Extinction rate estimates in particular should be interpreted cautiously, as they are potentially biased and often differ dramatically from true extinction rates in simulated phylogenies^47^. One of the reasons for this is BAMM’s assumption of no rate shifts on unobserved, extinct branches. Thus, we also estimated branch-specific diversification rates with RevBayes^51^, a similar approach which solves extinction-related caveats of BAMM by drawing diversification rates from a discrete, not continuous, distribution, allowing correct probability calculation across all possible rates. RevBayes output was visualised using the associated R package *RevGadgets* v.1.0.0 (https://github.com/revbayes/RevGadgets).

Finally, as an alternative to Bayesian methods, we ran a similar, maximum-likelihood analysis with MEDUSA^52^, implemented in the R package *geiger* v.2.0.6.4^53^. MEDUSA calculates the likelihood of obtaining a given tree with its particular phylogenetic (shape, branch lengths) and taxonomic (node age, extant species richness) properties, given specific values of birth rate (i.e. speciation) and death rate (i.e. extinction)^52^. Diversification rates are allowed to vary among clades, but are held constant through time, as opposed to BAMM. It identifies the likeliest birth and death rate values and calculates the model’s AIC score. It then proceeds to fit alternative, increasingly complex models of diversification to the phylogeny, calculating AIC scores of each. The process is stopped when a more complex model is no longer a significant improvement over the previous one^52^. Incomplete sampling was accounted for in the ‘richness’ file.

### Correlating candidate factors to macroevolutionary rates

We proceeded to test potential factors influencing nephilid speciation and extinction using state-dependent (SSE) models of diversification, all included in the R package *diversitree* v.0.9-13^54^ (see Supplementary Data online). We used BiSSE (Binary-state Speciation and Extinction Model)^55^ to estimate how speciation and extinction rates are affected by the state of a binary (two-state) character. In essence, BiSSE simultaneously models character change and the effect of this change on diversification. It acquires the probability that extant species would evolve the way they did, given a specific model of the character’s effect on evolution. Provided a phylogenetic tree and a binary character, BiSSE calculates this probability according to six parameters: speciation and extinction rates for state 0 and state 1, and rates of the two character state transitions. BiSSE applies maximum likelihood to estimate the parameters and with them tests the hypothesis that speciation and extinction rates do or do not depend on the state of the chosen character^55^. Because regular BiSSE assumes constant rates over time, we also applied the function *bisse.td*, which allows rates to change at a specified point in time.

We tested two binary traits for effects on nephilid diversification: island versus continental distribution and tropical versus subtropical distribution (the latter included two species with predominantly temperate distribution). We first estimated all six parameters in the ‘full model’, and then repeated the analyses with three types of constraints: equal speciation rates (λ_1_ = λ_0_), equal extinction rates (μ_1_ = μ_0_), and equal transition rates between states (q_10_ = q_01_). The full model was tested against each constrained model with ANOVA. Significant differences between them would suggest that macroevolutionary process do in fact depend on the state of the trait.

For time-dependent BiSSE testing island versus continental distribution, the age of the genus *Herennia*, which contains most cases of island endemism, was set as the point in time when rates were allowed to change (i.e. 26 mya). For tropical versus subtropical distribution, the approximate age of the oldest species, currently inhabiting subtropical or temperate climates, was set as the time switch point (i.e. 45 mya). For each trait, we created a *full* model, where speciation, extinction and state transition rates before and after the switch point were unconstrained. We then separately allowed each rate to vary across the two time periods, while keeping the other two rates constrained. The resulting three models and the *null* model (with all rates constrained across both time periods—in other words, the regular BiSSE model) were individually compared against the full model for improvement of fit.

BiSSE has been used in numerous studies and proved especially powerful for large trees, e.g.^56^. However, Rabosky and Goldberg^57^ point out that it is concerningly easy to obtain a statistically significant association between speciation rate and a quickly evolving neutral trait. They demonstrate this Type I error empirically and question the validity of conclusions made from BiSSE analyses in existing literature^57^.

To test the main proposed correlates of diversification, phenotype extremeness and dispersal propensity, we used QuaSSE (Quantitative-state Speciation and Extinction)^58^, the most complex of the SSE-class analyses, where speciation and extinction rates are modelled as functions of a continuous trait. It models and compares four alternative types of functions: constant, linear, sigmoidal and hump (modal) functions. We expect the latter to optimise as the best supported type of relationship between trait values and diversification.

We used body length, a standard in SSD research, as the tested extreme phenotype trait. We tested female and male body length separately, as well as body length SSD, calculated as the mean female body length divided by mean male body length for each species^16^. The phylogenetic tree used in the analysis for male body length and body length SSD omitted four species of *Herennia* (*H. gagamba*, *H. maj*, *H. milleri* and *H. oz*) for which the males are unknown.

To test the intermediate dispersal hypothesis, we used each species’ estimated area of occupancy (AOO) as a proxy for dispersal propensity, under the rationale that species with greater dispersal propensity will occupy and maintain presence on a larger geographical area. AOO is an established metric that records the area of suitable habitat, presently occupied by a selected species (after IUCN Standards and Petition Committee). AOO values were recovered from the IUCN Red List (https://www.iucnredlist.org/) and from unpublished data for *Herennia milleri*, *Herennia tone* and *Trichonephila edulis* (M. Kuntner and P. Cardoso, unpublished data). AOO values for species with single known specimens (*Herennia eva*, *H. maj* and *H. tsoi*) were arbitrarily assigned the minimal recorded nephilid AOO, that of *Nephilingis dodo*. Due to the extremely large variation in AOO values among species, we log-transformed the data prior to QuaSSE calculations.

Macroevolutionary literature has seen much debate on whether rates of extinction can be estimated from phylogenies lacking fossil data^30,48,55,59,60^. Cautious interpretation of extinction rate estimates is advised repeatedly and estimation is even discouraged when diversification is not uniform across the phylogenetic tree^59^. This is clearly the case in the nephilid phylogeny with a wide variation in species richness across genera, while the tree itself is small, reducing statistical power. Considering these limiting factors on top of QuaSSE’s calculation complexity, we kept the background extinction rate constant throughout the analysis and fitted the four candidate functions to speciation only. Additionally, we tested alternative versions of all four functions where drift (directional trend) is not constrained to zero. Positive values of the drift parameter signify an increase in the modelled trait with time, and negative values signify a decrease. All resulting QuaSSE models were compared with ANOVA and the difference in fit assessed via Akaike Information Criterion (AIC) values.

## Supplementary Information

Supplementary Information 1.

Supplementary Information 2.

Supplementary Information 3.

## Data Availability

All data generated and analysed during this study are included in this published article and its Supplementary Information and Supplementary Data files.
