# Supplementary material for: Exploring diversification drivers in golden orbweavers
Source: Sci Rep. 2021 Apr 29;11:9248. doi: 10.1038/s41598-021-88555-3 (PMC8084975; doi:10.1038/s41598-021-88555-3)
Supplement: Supplementary file 2 — Supplementary Information 2. [file 41598_2021_88555_MOESM2_ESM.docx]

**Exploring diversification drivers in golden orbweavers**

Eva Turk*, Simona Kralj-Fišer, Matjaž Kuntner

**Supplementary Figure S1:** Rate-through-time plot for rate of speciation in the nephilid phylogeny, inferred by BAMM.


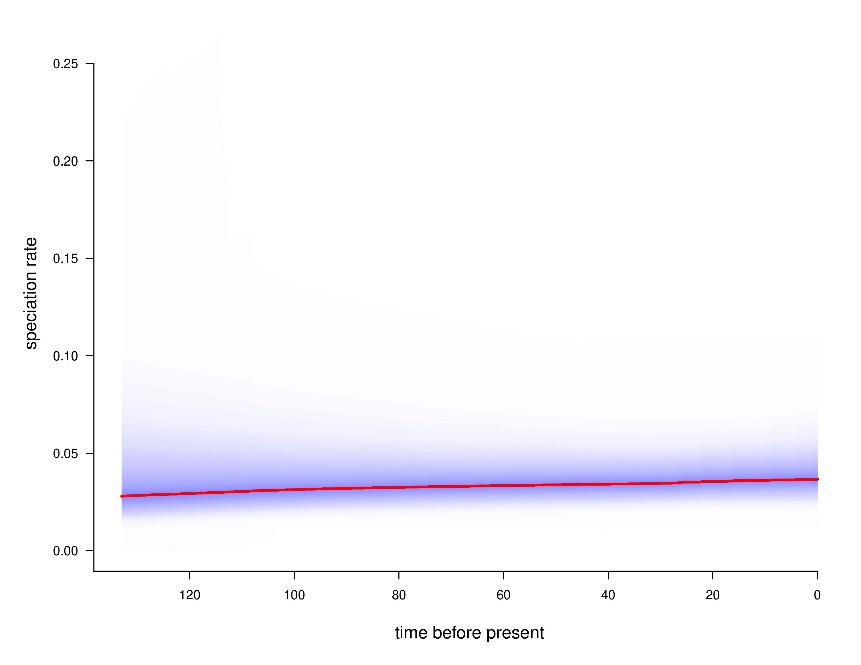


**Supplementary Figure S2:** Rate-through-time plot for rate of extinction in the nephilid phylogeny, inferred by BAMM.


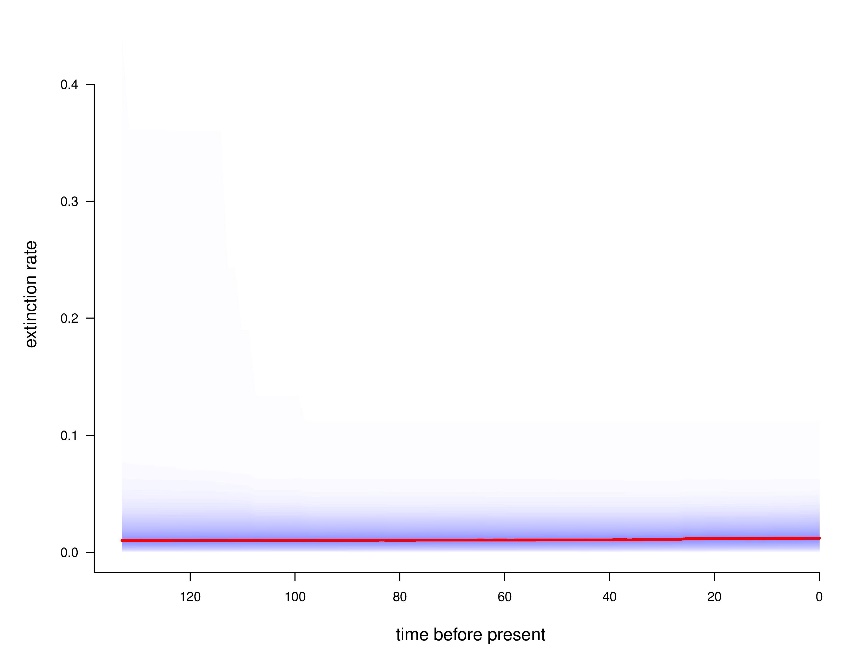


**Supplementary Figure S3:** Branch-specific diversification rates in the nephilid phylogeny, inferred by RevBayes.


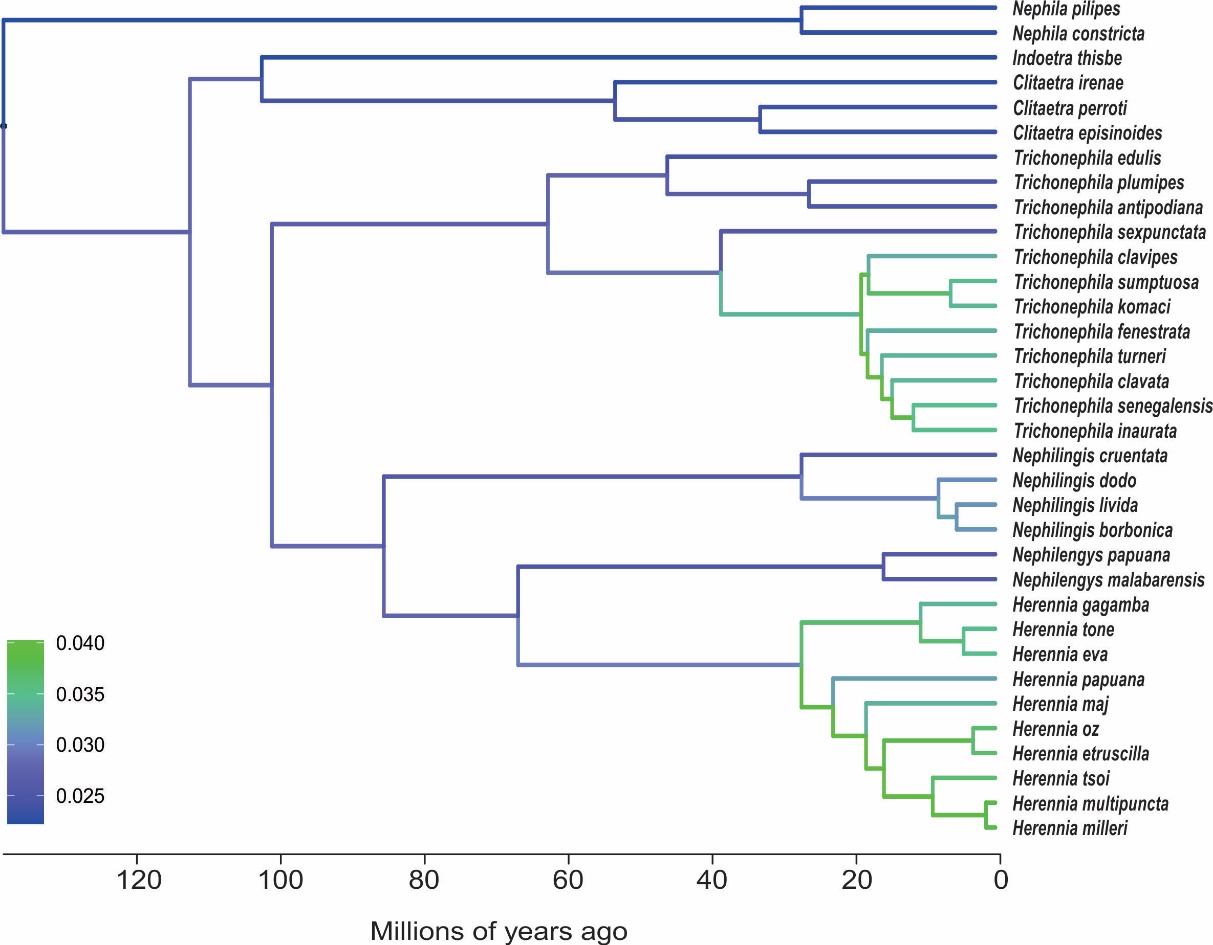


**Supplementary Table S1:** MuSSE model comparison with ANOVA. The analysis tests for an effect of a multi-state trait, web type, on speciation (λ), extinction (μ) and state transition (q). The asterisk signifies a marginally significant p-value (0.05 < p < 0.1).

| model | lnLik | AIC | Pr(>\|Chi\|) |
| --- | --- | --- | --- |
| minimal | -162.41 | 330.82 |  |
| free λ | -162.02 | 334.04 | 0.68 |
| free μ | -161.14 | 332.27 | 0.28 |
| free q | -158.79 | 329.58 | 0.06 * |

**Supplementary methods**

We applied MuSSE (Multi-State Speciation and Extinction)^1^ to test the correlation between diversification and web type, a trait with three distinct states: arboricolous (tree-hugging), hybrid (attached to substrate at the upper frame only) and aerial webs (fully suspended in the air column)^2^. Due to the dramatic difference in web architecture and anchoring point positioning of each web type, we only allowed for evolutionary transition between arboricolous and hybrid webs (q_12_ ↔ q_21_) and between hybrid and aerial webs (q_23_ ↔ q_32_), but not between arboricolous and aerial webs (q_13_ = q_31_ = 0). We created the ‘simple model’, where speciation rates, extinction rates and rates of transition were equal for all trait states (λ_1_=λ_2_=λ_3_, μ_1_=μ_2_=μ_3_, q_12_=q_21_=q_23_=q_32_). In separate models, we then allowed speciation, extinction, and trait state transition rates to vary. We tested each of these three models against the simple model using ANOVA to look for improvement in model fit.

**Supplementary results**

MuSSE recovers no improvement in model fit when speciation and extinction rates are allowed to vary among species with different types of webs, however, it recovers marginally significant improvement when transition rates between web types are allowed to vary (p=0.06; see Supplementary Table S1 online). The highest transition rate is recovered for hybrid to arboricolous webs (q_21_=0.0075), followed by hybrid to aerial webs (q_23_=0.0052). Rates of transition into hybrid webs from both other types are much lower (q_12_<0.0001, q_32_<0.0001).

**Supplementary discussion**

The MuSSE analysis finds only marginal support for model fit improvement when transition rates between web types are allowed to vary. Which transitions it supports most, though, is not entirely in accordance with web type transitions suggested by Kuntner et al.^2^. The highest transition rate, that from hybrid to arboricolous webs, is indeed found in Kuntner et al.^2^ at the split between the *Nephilengys* and *Herennia*. There is no instance of the second highest transition rate, from hybrid to aerial webs. The reverse transition, from aerial to hybrid webs, was recovered between *Trichonephila* and the *Nephilingis + Nephilengys + Herennia* clade, but this transition is poorly supported by our results. Kuntner et al.^2^ also suggest one transition from aerial to arboricolous, which is not allowed in our analysis. These results are inconclusive regarding their potential role in nephilid diversification. Still, considering the two most species rich genera, *Trichonephila* and *Herennia*, weave different types of webs, one could speculate webs do not play a notable role in speciation and extinction dynamics.

**References**

1. FitzJohn, R. G. Diversitree: Comparative phylogenetic analyses of diversification in R. *Methods Ecol. Evol.* **3**, 1084–1092 (2012).

2. Kuntner, M. *et al.* Golden orbweavers ignore biological rules: Phylogenomic and comparative analyses unravel a complex evolution of sexual size dimorphism. *Syst. Biol.* **68**, 555–572 (2019).
